# Improving Acceptability of mHealth Apps—The Use of the Technology Acceptance Model to Assess the Acceptability of mHealth Apps: Systematic Review

**DOI:** 10.2196/66432

**Published:** 2025-05-07

**Authors:** Ahmer Adnan, Rebecca Eilish Irvine, Allison Williams, Matthew Harris, Grazia Antonacci

**Affiliations:** 1 Faculty of Medicine Imperial College London London United Kingdom; 2 National Institute of Health Research (NIHR) Applied Research Collaboration (ARC) Northwest London Department of Primary Care and Public Health Imperial College London London United Kingdom; 3 Centre for Health Economics and Policy Innovation (CHEPI) Business School Imperial College London London United Kingdom

**Keywords:** technology acceptance model, mHealth, mobile health apps, health information technology acceptance model

## Abstract

**Background:**

Mobile health apps (MHAs) are increasingly used in modern health care provision. The technology acceptance model (TAM) is the most widely used framework for predicting health care technology acceptance. Since the advent of this model in 1989, technology has made generational advancements, and extensions of this model have been implemented.

**Objective:**

This systematic review aimed to re-examine TAM models to establish their validity for predicting the acceptance of modern MHAs, reviewing relevant core and extended constructs, and the relationships between them.

**Methods:**

In this systematic review, MEDLINE, Embase, Global Health, APA PsycINFO, CINAHL, and Scopus databases were searched on March 8, 2024, with no time constraints, for studies assessing the use of TAM-based frameworks for MHA acceptance. Studies eligible for data extraction were required to be peer-reviewed, English-language, primary research articles evaluating MHAs with health-related utility, using TAM as the primary technology acceptance evaluation framework, and reporting app use data. Data were extracted and grouped into 5 extended TAM construct themes. Quality assessment was conducted using the Joanna Briggs Institute (JBI) tools. For cross-sectional methodologies (9/14, 64%), the JBI checklist for analytical cross-sectional studies was used. For non–cross-sectional studies (5/14, 36%), the JBI checklist most relevant to the specific study design was used. For mixed methods studies (1/14, 7%), the JBI checklist for qualitative studies was applied, in addition to the JBI checklist most suited to the quantitative design. A subsequent narrative synthesis was conducted in line with PRISMA (Preferred Reporting Items for Systematic Reviews and Meta-Analyses) methodology.

**Results:**

A total of 2790 records were identified, and 14 were included. Furthermore, 10 studies validated the efficacy of TAM and its extensions for the assessment of MHAs. Relationships between core TAM constructs (perceived usefulness, perceived ease of use, and behavioral intention) were validated. Extended TAM constructs were grouped into 5 themes: health risk, application factors, social factors, digital literacy, and trust. Digital literacy, trust, and application factor extended construct themes had significant predictive capacity. Application factors had the strongest MHA acceptance predictive capabilities. Perceived usefulness and extended constructs related to social factors, design aesthetics, and personalization were more influential for those from deprived socioeconomic backgrounds.

**Conclusions:**

TAM is an effective framework for evaluating MHA acceptance. While original TAM constructs wield significant predictive capacity, the incorporation of social and clinical context-specific extended TAM constructs can enhance the model’s predictive capabilities. This review’s findings can be applied to optimize MHAs’ user engagement and minimize health care inequalities. Our findings also underscore the necessity of adapting TAM and other acceptability frameworks as the technological and social landscape evolves.

**Trial Registration:**

PROSPERO CRD42024532974; https://www.crd.york.ac.uk/PROSPERO/view/CRD42024532974

## Introduction

Rapid advancements in technology have transformed health care, from the widespread implementation of electronic health records and minimally invasive robotic surgery to artificial intelligence assisting with numerous clinical activities [1,2]. Ultimately, technology helps reduce physician workloads and improve patient outcomes, all while maintaining cost-effectiveness for health care systems [3,4].

With this relentless technological evolution and the integration of various health monitoring capabilities in mobile devices, mobile health (mHealth) apps (MHAs) are becoming even more accessible [5]. MHAs are software implementations that process health-related data and provide health-related functionality, accessible via mobile devices [5,6]. In recent years, the use of MHAs has grown exponentially, becoming prevalent among both the general population and health care professionals (HCPs) [5]. MHAs promise to enhance health care outcomes by improving access, engagement, and efficiency of health care services [7].

MHAs possess numerous clinical uses. Common functions include reminders, notifications, activity tracking, and tailored information [5,7,8]. Many MHAs combine multiple functionalities, optimizing their utility and efficacy [9]. Not only do MHAs carry substantial utility for the delivery of clinical care, but they also host a range of diverse functionalities applicable to clinical research. For example, journaling capabilities can be used to record episodes of angina, reducing recall bias, substantially improving the validity of clinical trials exploring cardiac parameters [10]. The success and sustainability of these technologies critically depends on their acceptability to users, both patients and HCPs alike.

Developed in 1989 by Davis [11], the technology acceptance model (TAM) is the most widely used framework to predict technology acceptance and uptake, especially for health care technologies [11-13]. TAM focuses on two fundamental principles: perceived usefulness (PU) and perceived ease of use (PEOU). PU proposes that app use depends on the extent a user finds it beneficial to their desired outcome [11]. Contrastingly, PEOU suggests that the difficulty of using an app may outweigh potential performance benefits, reducing uptake and use, hence, apps requiring less effort to use will have greater user engagement [11]. As such, PU and PEOU are inherently interdependent and vary between users.

The relationship between these constructs and technology acceptance becomes more complex in health care settings. For instance, PU may increase with the severity of a disease, as individuals with serious diagnoses might find MHAs more useful. However, some studies suggest that those who are more unwell are less likely to adopt MHAs [14]. This can be partially explained by the “Healthy User Effect” where healthier individuals are more likely to be health-conscious and engage with health-promoting technologies like MHAs [14-16]. Therefore, the traditional predictive power of PU in this context may be diluted [14-16].

Since its inception, TAM has undergone incremental development and refinement to better predict technology acceptance. In 2000, TAM2 introduced new constructs related to social influence processes and cognitive instrumental processes, such as subjective norms and output quality [17]. Then in 2008, TAM3 included a series of anchors and adjustments accounting for increasing understanding and penetration of modern technologies into the market [18]. There are also technology-specific models, such as the health information TAM proposed in 2012, which integrates principles of the Health Belief Model into TAM, adding the perceived threat construct, composed of perceived susceptibility and perceived seriousness [19].

Apple launched the first iPhone just months prior to TAM3 in early 2008 [18,20]. With technology continuing to advance at an exponential pace [21], it is crucial to re-evaluate TAM and its extensions, their applicability, and their efficacy. Mobile devices dominate the market, with each new generation more powerful and easier to use than the last, thus improving consumer familiarity and digital expertise. Consequently, the PEOU and PU environment has been completely redefined since the introduction of TAM. As technology use has expanded, so too has public concern for data security and consumer trust, likely directly influencing technology acceptance, especially for MHAs due to the sensitivity of data shared [12,22].

Few reviews have evaluated the health care applications of TAM, with even fewer assessing TAM within the context of MHAs [23-25]. These studies have largely validated TAM’s ability to assess mobile health (mHealth) technology acceptance [23-25]. Most studies do not assess actual use (AU) of the technology. Instead, they report behavioral intention (BI), which, while strongly correlated with AU, is not a perfect predictor [23-26]. Furthermore, many systematic reviews employ restrictive protocols and are outdated. Technology has evolved substantially, as has the literature base and geopolitical situation of the world, which will undoubtedly affect technology acceptance [23-25].

This systematic review seeks to re-evaluate the applicability and efficacy of TAM and its extensions. We aim to explore whether these models still provide a robust framework for predicting the acceptability of MHAs amid changing user expectations, technical capabilities, and heightened data security concerns. Furthermore, this review will highlight the most influential extended TAM constructs for MHA assessment and significant relationships between core and extended constructs. This reassessment is crucial for informing the development and equitable implementation of MHAs that are not only technologically advanced, but also widely used by health care providers and patients.

## Methods

### Search Strategy

This systematic review was conducted following a PROSPERO-registered protocol (CRD42024532974) and PRISMA (Preferred Reporting Items for Systematic Reviews and Meta-Analyses) guidelines (Multimedia Appendix 1) [27,28]. A systematic literature search was performed on March 8, 2024. MEDLINE, Embase, APA PsycInfo, Global Health, CINAHL, and Scopus databases were searched from database inception to the search date. The search was structured using a combination of subject headings and keywords related to the “technology acceptance model” and “mobile health applications.” A specialist librarian assisted in search strategy development (Multimedia Appendix 2).

### Study Inclusion and Exclusion Criteria

Only primary studies published in peer-reviewed English-language papers that evaluated MHAs with health- or clinical management–related utility, structured their acceptance model primarily on TAM, and recorded MHA AU data were included (Textbox 1). MHAs targeted primarily at HCPs for education or as hospital information systems (such as electronic health records) were excluded, as the design priorities, usability parameters, and implementation objectives for MHAs differ significantly between HCP-focused and patient-focused apps. HCP-focused MHAs serve professional users and focus on improving clinical education, decision-making, or administrative tasks. These apps prioritize clinical accuracy, interoperability with health care systems, and adherence to regulatory standards. Contrastingly, patient-focused MHAs aim to empower individual users by improving health literacy, promoting behavior change, and enhancing engagement. They prioritize features such as accessibility, personalization, gamification, and user-friendliness. Including both types of apps could confound the analysis, as their usability and acceptability metrics are inherently different. By focusing on patient-centered MHAs, this review ensures actionable insights that are directly applicable to improving patient care and outcomes.

**Inclusion and exclusion criteria.**

**Inclusion criteria**

- Primary studies published in English
- Technology acceptance framework primarily based on the technology acceptance model
- Assessment of mobile health app (MHA) actual use
- Assessment of MHAs with clinical or health-related utility

**Exclusion criteria**

- MHAs targeted primarily at health care professionals for education or as hospital information systems (such as electronic health records)
- Secondary literature such as reviews, letters, protocols, and editorials

### Screening

After primary deduplication with EndNote 21 (Clarivate), record screening and data extraction was conducted using Covidence (Veritas Health Innovation) [29,30]. AA conducted the primary screening and full-text review of the entire dataset. To ensure complete dual review, AW and REI independently assessed 25% and 75% of the dataset, respectively, ensuring that each study was screened by 2 authors. Any conflicts were further discussed with a third author to resolve the decision.

### Study Quality Assessment and Data Extraction

Quality assessment and risk of bias evaluation was conducted synchronously with data extraction. Joanna Briggs Institute (JBI) critical appraisal tools were used for quality assessment [31]. The JBI checklist for analytical cross-sectional studies was used for cross-sectional methodologies (9/14, 64%). For non–cross-sectional methodologies (5/14, 36%), the JBI checklist specific to the study design was used. For mixed methods studies (1/14, 7%), the JBI checklist for qualitative studies was applied to assess the qualitative aspect, in addition to the JBI tool most suited to the quantitative design being used to assess the quantitative arm. Any studies found not to be robust upon quality appraisal, for example, due to substantial sources of bias, unrepresentative sampling, unclear inclusion and exclusion criteria, incomplete data reporting, inappropriate statistical techniques, or inadequate methodological explanation, would be flagged for discussion and potential exclusion. However, in this review, no studies were excluded based solely on quality appraisal.

A tailored data extraction template was developed to collect study details across 4 domains—metadata and study context, details on the TAM model used, study methodology, and key study findings, including relevant constructs and their relationships (Multimedia Appendix 3). Quality appraisal and data extraction were conducted by 2 authors (AA and REI). Any conflicts were resolved by review and agreement of a third author. All authors had complete access to all data.

### Data Synthesis

Subsequently, a narrative synthesis was conducted to present the key findings from the literature. Thematic analysis of TAM constructs, following Braun and Clarke’s [32] framework, was conducted to aid in categorization and theme-specific analysis. This approach helped to account for construct heterogeneity between studies [32]. Themes were defined inductively, based on both standard and data-specific concepts, with TAM constructs coded and then systematically grouped by theme.

## Results

### Study Selection

**Figure 1:**
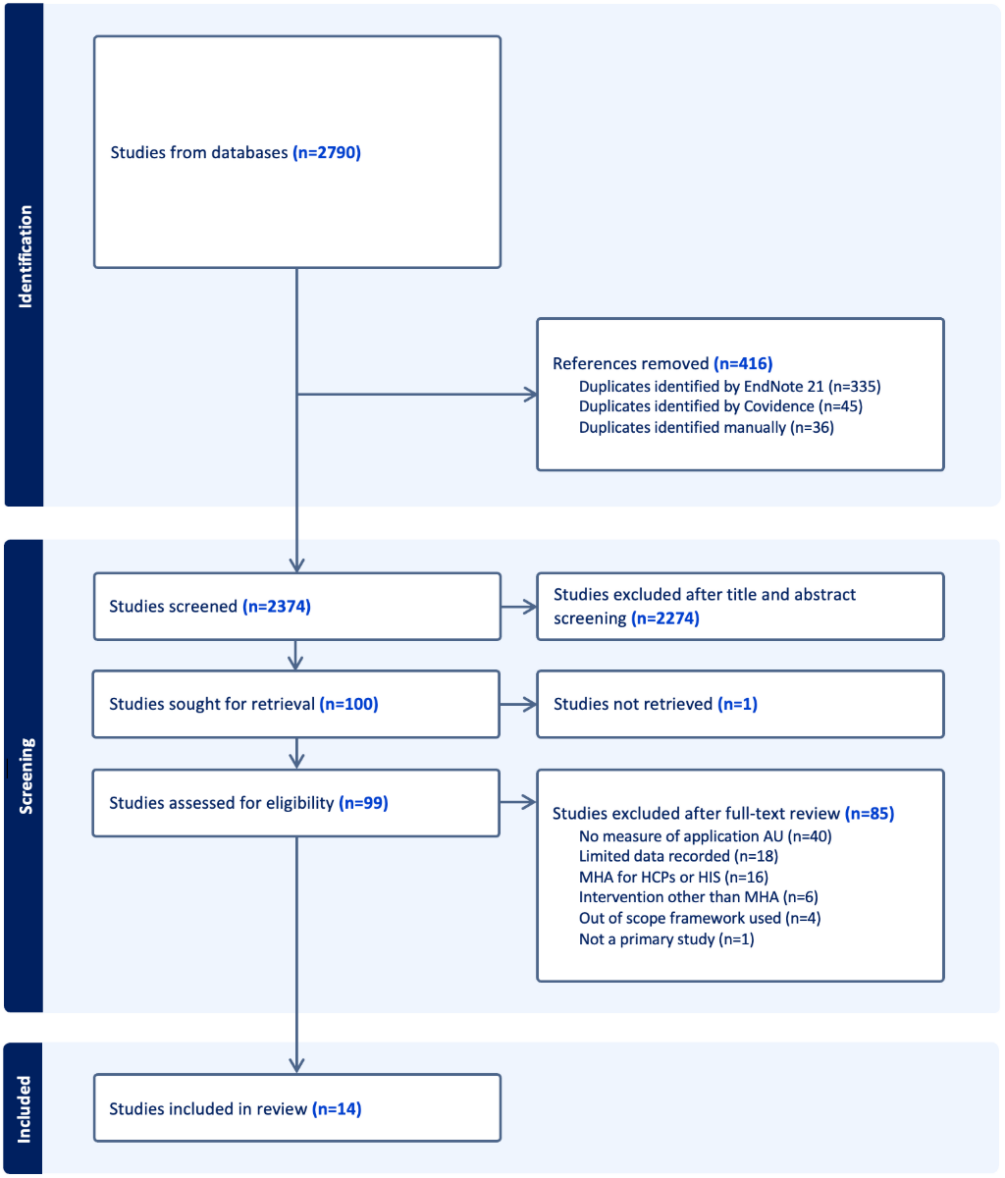
PRISMA diagram demonstrating the study selection and screening process. AU: actual use; HCP: health care professional; HIS: hospital information system; MHA: mobile health app.

A total of 2374 unique references were identified. Of these, 14 studies met the inclusion criteria and were included for data extraction [33-46]. Study selection is outlined in Figure 1.

### Study Characteristics

Study characteristics are shown in Table 1. The 14 studies included in this review have been conducted across 9 countries. The United States hosted most of the studies (n=5) [38-40,44,45]. In total, 11 studies were conducted in high-income countries [35-40,42-46], 3 in upper-middle income countries [33,34,41], and 1 in a low-middle income country (LMIC) [34]. Of note, Ali et al [34] recruited participants in China (upper-middle income country) and Pakistan (LMIC). No study was conducted exclusively in LMICs or low-income countries (LICs).

Most studies were of cross-sectional design (n=9) [33,35-40,45,46] and quantitative in nature (n=13) [33-35,37-46]. Furthermore, 5 were prospective and longitudinal studies [34,41-44], and there was 1 mixed methods study [36]. The large majority of studies recruited participants in the community setting (n=11) [33-37,39,40,42,43,45,46] with only Byrd et al [38], Dou et al [41], and Louissant et al [44] investigating secondary care. In addition, 5 papers recruited only university students and staff [34,35,39,43,46], which may introduce selection bias. An equal number of studies used self-reported AU (n=7) [35-37,39,40,45,46] as used app usage logs (n=7) [33,34,38,41-44], which is a more robust method of assessing the AU of MHAs, eliminating potential recall bias [10,48]. Furthermore, most studies used validated TAM questionnaires derived from the literature (n=11) [33,35-39,41-45], minimizing the effects of measurement bias.

A total of 10 studies evaluated the acceptance of MHAs for specific clinical domains [33-36,38,41-45], such as hypertension [41] or smoking cessation [34], and 4 evaluated the general acceptability of all MHAs used by participants [37,39,40,46].

**Table 1:** Study characteristics.

Study ID	Country, World Bank classification [47]	Study design	Total number of participants	Health care setting	Clinical domain	App
Akdur et al [33]	Turkey, UMIC^a^	Quantitative, cross-sectional study	658	Community	Dietetics	Diyetkolik (PCI Yazilim Danismanlik ve Organizasyon Ltd)
Ali et al [34]	China and Pakistan, UMIC and LMIC^b^	Prospective, quantitative, longitudinal study	581 Chinese and 639 Pakistani	Community	Smoking cessation	Smokers Mirror (Mr Muhammad Hassan Nasir, Eocean Pvt Ltd) and QR code for smoking cessation
Alsyouf et al [35]	Saudi Arabia, HIC^c^	Quantitative, cross-sectional study	586	Community	Exposure detection (COVID-19)	Tabaud app (SDAIA^d^ NIC^e^ in collaboration with Saudi MOH) and Tawakkalna app (SDAIA)
Balki et al [36]	United Kingdom, HIC	Mixed methods, cross-sectional study	25	Community	Social isolation	Numerous
Bao and Lee [37]	Singapore, HIC	Quantitative, cross-sectional study	906	Community	All MHAs^f^	Numerous
Byrd et al [38]	United States, HIC	Quantitative, cross-sectional study	1254	Secondary care center	Communication	Vocera (Vocera Communications Inc)
Cho et al [39]	United States, HIC	Quantitative, cross-sectional study	408	Community	All MHAs	Numerous
Cramer et al [40]	United States, HIC	Quantitative, cross-sectional study	332	Community	All MHAs	Numerous
Dou et al [41]	China, UMIC	Prospective, quantitative, longitudinal study	152	Secondary care center	Hypertension	Blood Pressure Assistant (Biomedical Informatics Laboratory, Zhejiang University)
Hurmuz et al [42]	Netherlands, HIC	Prospective, quantitative, longitudinal study	72	Community	Falls prevention	Stranded (RRD^g^ in collaboration with University of Twente)
Jeon and Park [43]	Republic of Korea, HIC	Prospective, quantitative, longitudinal study	94	Community	Weight management	Proprietary app
Louissaint et al [44]	United States, HIC	Prospective, quantitative, longitudinal study	102	Secondary care center	Cirrhosis	Encephal app (Dr Jasmohan S Bajaj, Virginia Commonwealth University, and McGuire VA Medical Center)
McKee et al [45]	United States, HIC	Quantitative, cross-sectional study	2619	Community	Psychology	Numerous
Shemesh and Barnoy [46]	Israel, HIC	Quantitative, cross-sectional study	168	Community	All MHAs	Numerous

^a^UMIC: upper middle-income country.

^b^LMIC: lower middle-income country.

^c^HIC: high-income country.

^d^SDAIA: Saudi Data and Artificial Intelligence Authority.

^e^NIC: National Information Center.

^f^MHA: mobile health app.

^g^RRD: Roessingh Research and Development.

### TAM Constructs: Core and Extended

**Figure 2:**
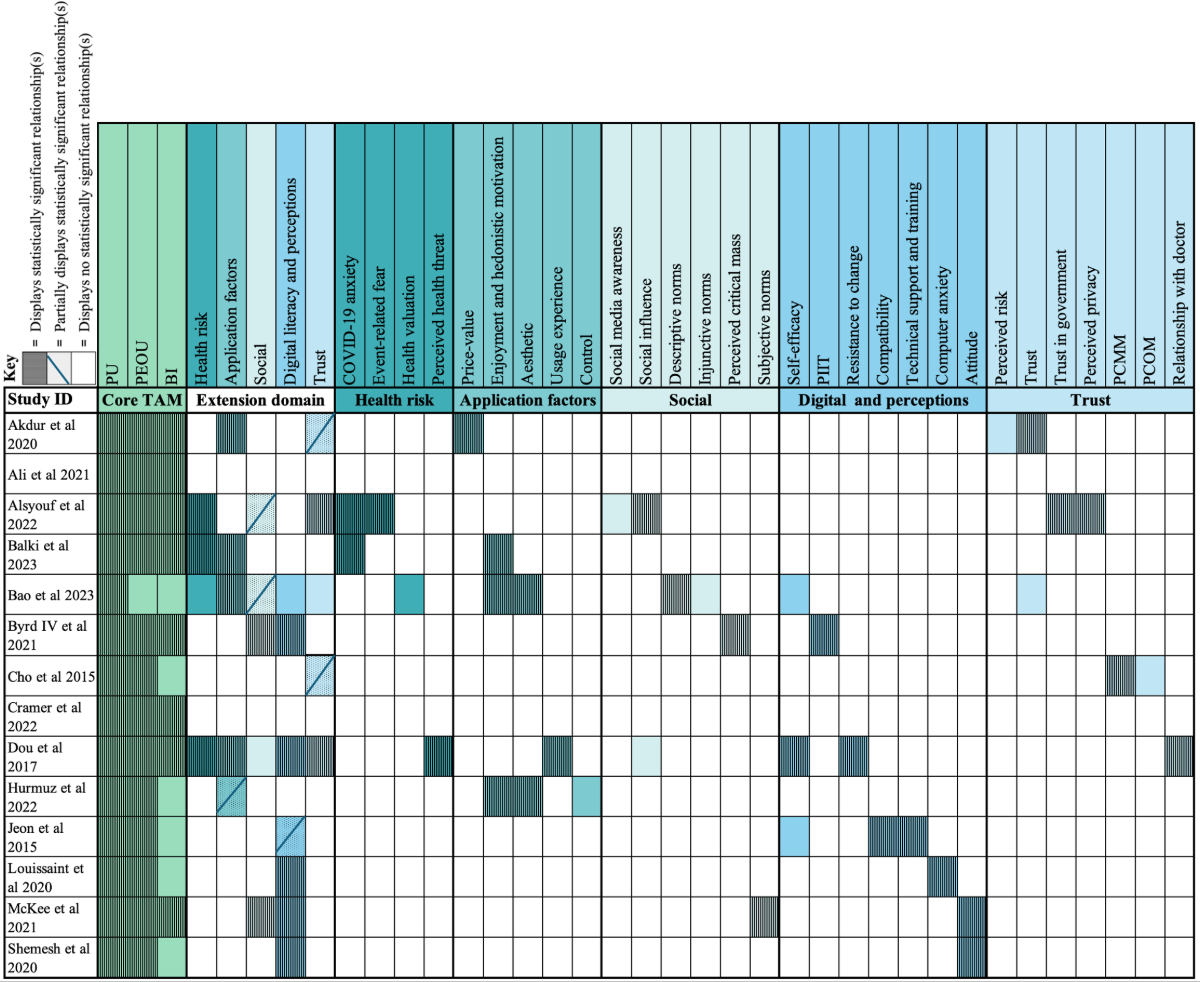
Core and extended technology acceptance model construct use across studies. Key is available in the figure and refers to colored squares; uncolored squares depict that the parameter was not investigated. BI: behavioral intention; PCMM: perceived credibility of mass media; PCOM: perceived credibility of online media; PEOU: perceived ease of use; PIIT: personal innovativeness in the domain of information technology; PU: perceived usefulness; TAM: technology acceptance model.

All studies structured their MHA acceptance model around TAM. Multimedia Appendix 3 presents the overall findings of each study. All included studies used the 3 core TAM constructs (PU, PEOU, and BI) [33-46]. Of these, 2 studies focused solely on the 3 core TAM constructs without incorporating extended constructs [34,40]. The other 12 studies used extended models with additional constructs (Figure 2) [33,35-39,41-46]. Extended constructs have been categorized by theme. Extended construct themes were (1) health risk [35-37,41], (2) application factors [33,36,37,41,42], (3) social factors [35,37,38,41,45], (4) digital literacy and perceptions [37,38,41,43-46], and (5) trust [33,35,37,39,41] (Figure 2, Table 2). Studies may have multiple extended constructs, each of which lies in a different theme; hence, studies may be in more than 1 theme (Figure 2, Table 2).

**Table 2:** Categorization of papers by extended TAM construct theme.

Study			Extended constructs		Findings
**Health risk constructs**					
	Alsyouf et al [35]	COVID-19 anxiety, event related fear		COVID-19 anxiety had a mediating role between other parameters. Event-related fear was found to be a significant mediator and acted on AU^a^ via influence of COVID-19 anxiety.	
	Balki et al [36]	COVID-19 anxiety		Significant predictive value. Negative relationship between COVID-19 anxiety and PU^b^, PEOU^c^, BI^d^, and AU. The pandemic, while increasing COVID-19 anxiety, which had detrimental impacts on PU and PEOU, led to the increase of digital communication technology use by 76%.	
	Bao and Lee [37]	Health valuation		No association between health valuation and BI and AU, however, the relationship with PEOU and PU was not investigated in this study	
	Dou et al [41]	Perceived health threat		Significant predictor of acceptance, mediating the BI via significant positive relationships with PU and PEOU.	
**Application factor constructs**					
	Akdur et al [33]	Price-value		Price-value was significant and had relationships with BI and PU.	
	Balki et al [36]	Perceived enjoyment		Perceived enjoyment was a strong predictor of BI and impacted all variables PU, PEOU, BI and AU. PE^e^ had a negative relationship with COVID-19 anxiety.	
	Bao and Lee [37]	Design aesthetic, hedonistic motivation		Design aesthetic associated with BI but not AU. Hedonistic motivation associated with BI and AU. Design aesthetic was found to close the gap caused in MHA^f^ usage due to education. Hedonic motivation’s association with BI and AU was the strongest overall.	
	Dou et al [41]	Usage experience		Usage experience had strong relationship with PEOU but not PU.	
	Hurmuz et al [42]	Enjoyment, aesthetic, and control		Enjoyment had a strong effect on PU. Aesthetics had a strong effect on PEOU. Control was insignificant.	
**Social constructs**					
	Alsyouf et al [35]	Social media awareness, social influence		Social media awareness had no statistically significant pathways. Social influence had a statistically significant, however, relatively weak predictive strength through indirect pathways.	
	Bao and Lee [37]	Descriptive norms, injunctive norms		Descriptive norms were found to be significantly associated with AU. Injunctive norms had no significant relationship with BI or AU. Combined, both only explained 2.41% of the variance in AU. Both constructs had a moderating relationship with education.	
	Byrd et al [38]	PCM^g^		PCM had significant influences on PU, PEOU, and BI.	
	Dou et al [41]	Social influence		Social influence had no effect on PU and was excluded as an insignificant construct.	
	McKee et al [45]	Subjective norms		In a TRA^h^ model, subjective norms had significant effects on BI and AU, when this was combined with TAM^i^, subjective norms were associated with PU and PEOU, although, overall, this model explained less variance in AU and model fit was not as unanimous among the tests as TAM or TRA alone. In the combined TAM and TRA model, the strongest relationship was between subjective norms and PU.	
**Digital literacy and perceptions constructs**					
	Bao and Lee [37]	Self-efficacy		Collected data but did not include it in the analysis beyond measures of central tendency.	
	Byrd et al [38]	PIIT^j^		PIIT had a small effect on PEOU and BI, but not PU.	
	Dou et al [41]	Self-efficacy, RTC^k^		Strong positive effects of self-efficacy on PEOU. RTC had a significant negative relationship with BI and PU, and was negatively influenced by the RWD^l^ construct.	
	Jeon and Park [43]	Self-efficacy, compatibility, technical support and training		Self-efficacy does not have a significant relationship with PU or PEOU, but is directly affected by compatibility, training, and technical support, hence is not relevant in a model assessing technology acceptance with no direct or indirect pathways affecting BI or AU. Compatibility positively influenced PU, PEOU, and BI. Technical support and training positively affected PEOU.	
	Louissant et al [44]	Computer anxiety		The computer anxiety construct was found to be integral to MHA download and was reduced by the presence of a caregiver as well as increased by age. It also had direct effects on PEOU and BI.	
	McKee et al [45]	Attitude		Attitude was found to be influenced by PU and PEOU while directly impacting BI in the TAM model.	
	Shemesh and Barnoy [46]	Attitude		Attitude was a significant factor. Subgroup analysis stratified by users and nonusers of MHA found attitudes to be the single construct which was significant in both groups (with PU, PEOU, and BI being insignificant in the non-users group).	
**Trust and privacy constructs**					
	Akdur et al [33]	Perceived risk, trust		Perceived risk showed no significant associations, attributing this to excessive scope of the construct. Trust had a statistically significant relationship with BI.	
	Alsyouf et al [35]	Trust in government, perceived privacy		Trust in government had significant relationships with AU moderated by social influence. Perceived privacy had a significant positive relationship with BI.	
	Bao and Lee [37]	Trust		No significant relationships between trust and BI or AU, attributing this to the regional policies and the trust citizens have in the data security policies.	
	Cho et al [39]	PCMM^m^, PCOM^n^		PCMM had statistically significant effects on PU and PEOU. PCOM had no statistically significant relationships.	
	Dou et al [41]	RWD		RWD helped reduce RTC which in turn promoted BI. RWD directly influenced PEOU and PU.	

^a^AU: actual use.

^b^PU: perceived usefulness.

^c^PEOU: perceived ease of use.

^d^BI: behavioral intention.

^e^PE: perceived enjoyment.

^f^MHA: mobile health app.

^g^PCM: perceived critical mass.

^h^TRA: theory of reasoned action.

^i^TAM: technology acceptance model.

^j^PIIT: personal innovativeness in the domain of information technology.

^k^RTC: resistance to change.

^l^RWD: relationship with doctor.

^m^PCMM: perceived credibility of mass media.

^n^PCOM: perceived credibility of online media.

**Figure 3:**
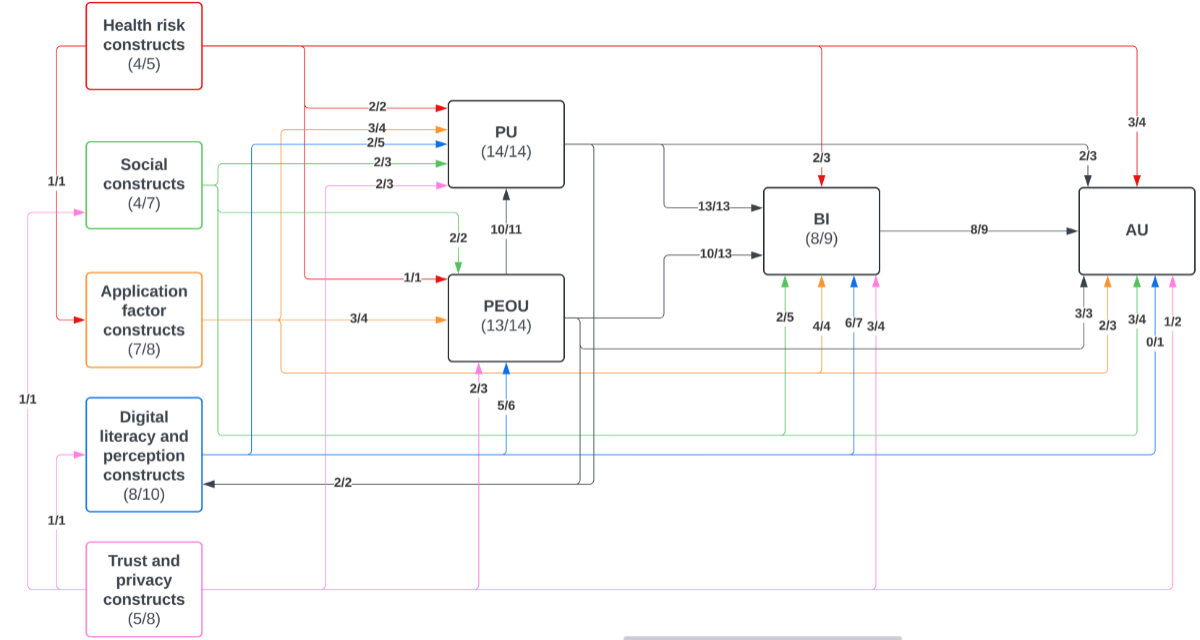
Diagram showing all statistically significant relationships between core technology acceptance model constructs and extended construct domains. Relationships and constructs shown by number of studies that found it to be statistically significant/total number of studies investigating the parameter, for example, in 9 studies investigating the relationship between behavioral intention and actual use, 8 found it to be statistically significant. AU: actual use; BI: behavioral intention; PEOU: perceived ease of use; PU: perceived usefulness.

**Figure 4:**
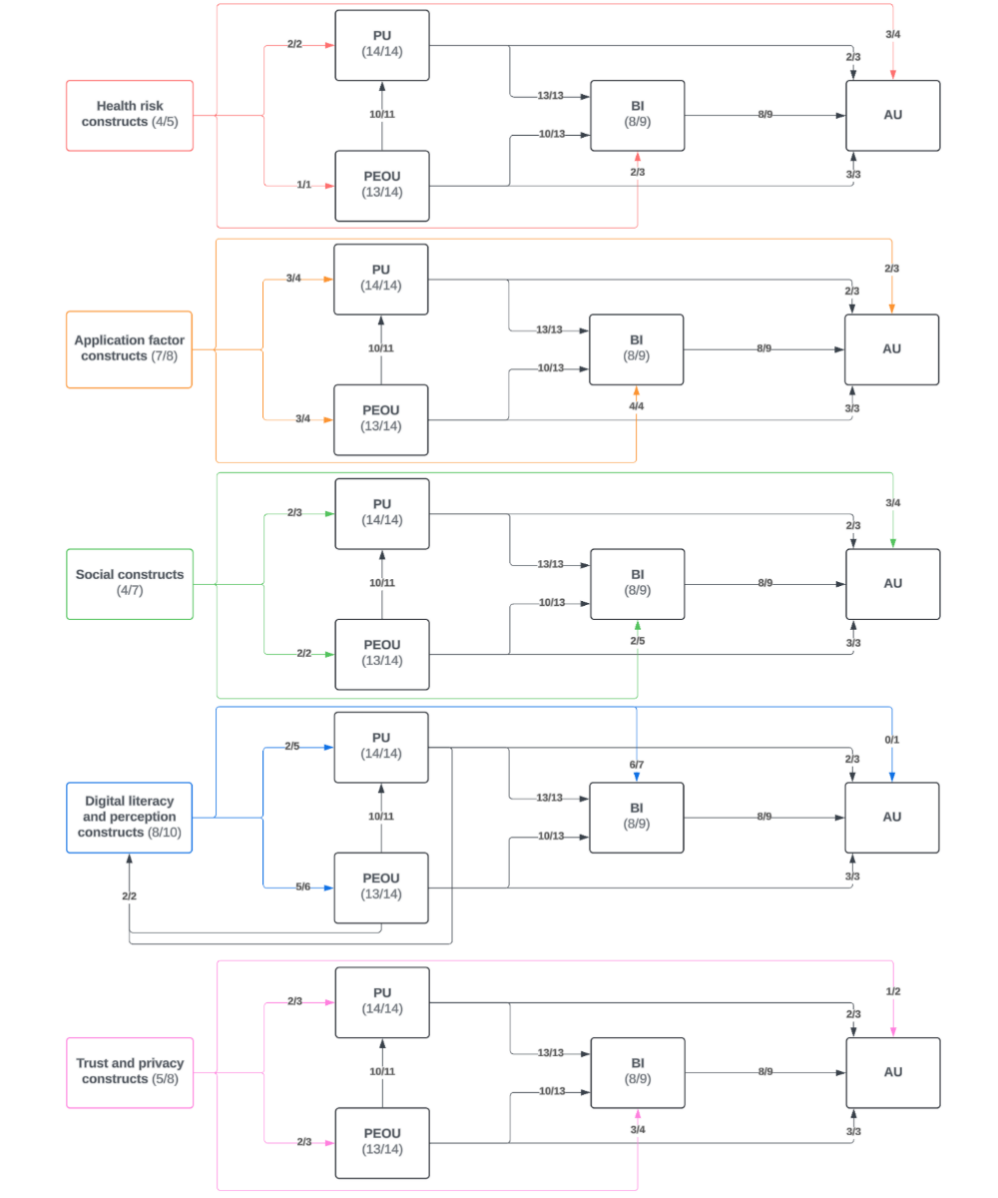
Relationship between core technology acceptance model constructs—perceived usefulness, perceived ease of use, behavioral intention, and actual use—and extended technology acceptance model construct themes. Relationships and constructs shown by number of studies that found it to be statistically significant/total number of studies investigating the parameter; for example, in 9 studies investigating the relationship between behavioral intention and actual use, 8 found it to be statistically significant. AU: actual use; BI: behavioral intention; PEOU: perceived ease of use; PU: perceived usefulness.

Most studies (n=10) validated TAM’s significant predictive power to assess technology acceptance [33-40,43,46]. This consistent validation demonstrates TAM’s robustness as a framework for predicting MHA adoption, particularly in diverse health care settings. In total, 13 studies validated the relationship between PU and BI (Figures 3 and 4) [33-45], and 10 validated the relationship between PEOU and BI [33-36,38-40,43-45]. Many studies (n=10) found that PEOU directly influenced PU [33-36,38-41,43,45]. Furthermore, 8 studies validated the relationship between BI and AU [33-36,38,40,41,45], with Dou et al [41] finding a statistically significant but weak relationship. McKee et al [45] investigated 3 different models and found a strong, significant relationship between BI and AU in their TAM and theory of reasoned action models. However, in their combined model, similar to Dou et al [41], this relationship was weak [45]. These findings suggest that the addition of extended constructs to TAM may sometimes dilute its predictive power, highlighting the importance of context-specific adaptations. Many other studies did not investigate the relationship between BI and AU (n=5) [37,39,42,43,46]. Only 1 study found a statistically insignificant relationship between BI and AU, although this team found that BI was significantly related to agreement to download the MHA under investigation [44].

The 2 studies exclusively examining the original TAM model were heterogeneous in study design. Both found TAM to be a good predictor of AU, validating the originally proposed TAM construct relationships (between PU, PEOU, and BI) [34,40]. Cramer et al [40] found PEOU was the strongest predictive construct with both direct and indirect effects on BI. Ali et al [34] found that PU was the strongest construct.

Overall, this review demonstrates that TAM’s core constructs are robust and versatile predictors of technology acceptance validated across diverse settings. Incorporating extended constructs tailored to specific contexts and demographics enhances its predictive power (Figures 2-4). However, the integration of extended constructs into TAM requires careful consideration of context, population, and application-specific features to maintain its predictive strength and relevance.

### Health Risk Constructs

In total, 4 studies examined 5 health risk constructs [35-37,41]. Of these, 4 constructs were found to have statistically significant relationships with direct or indirect contributions in the models assessed (Figure 2, Table 2) [35,36,41]. In addition, 2 constructs evaluated COVID-19 anxiety-related parameters and 2 examined factors related to general health concerns, with direct or indirect contributions in the models assessed (Figure 2, Table 2) [35,36,41].

Overall, health risk constructs, such as COVID-19 anxiety, perceived health threat, and event-related fear, were significant predictors of technology acceptance. Although the “health valuation” construct was not statistically significant, this may reflect methodological limitations in examining indirect pathways [37].

### Application Factor Constructs

Application factors played a pivotal role in influencing user engagement. In total, 5 studies evaluated application factor constructs [33,36,37,41,42], and 7 constructs had significant relationships [33,36,37,41,42]. 4 constructs centered on enjoyment and user experience, and all were deemed significant positive predictors [36,37,41,42]. 2 constructs evaluated the effects of MHA aesthetics, which were significant [37,42] and found to directly improve PEOU [42], while reducing the gap created by education disparities—an indirect indicator of socioeconomic status (SES) [37]. Largely driven by enjoyment and aesthetics, usage experience significantly impacted PEOU as well [41]. Price value was also significant [33].

### Social Constructs

Few social constructs were deemed statistically significant (n=4) [35,37,38,45]. Furthermore, 2 studies found social influence had poor predictive capacity and acted only via indirect pathways [35] or that it was not statistically significant [41]. Interestingly, 1 study discovered that lower SES groups (indicated by education status) were more influenced by social constructs [37]. These results suggest that social constructs may play a more significant role in influencing technology acceptance among lower SES groups, highlighting the need for targeted interventions in these populations.

### Digital Literacy and Perceptions Constructs

Digital literacy and perception constructs were integral to technology acceptance [38,41,43-46] (Table 2). In total, 8 constructs were seen to improve acceptance directly or indirectly, impacting numerous core TAM constructs (Table 2). Jeon and Park [43] found that the provision of technical support and training was effective in predicting and promoting technology acceptance. This construct likely has significant effects on core and extended constructs included in many models, such as self-efficacy, resistance to change, and computer anxiety.

### Trust and Privacy Constructs

Trust and privacy constructs varied in importance to extended TAM models (Table 2). Trust was deemed to be an important construct theme and took various forms (Figure 2, Table 2) [33,35,39]. One study disputed the importance of trust; however, its findings may be attributed to regional factors [37]. The risk of data breaches and other MHA-related risks was deemed not to be a predictive factor by Akdur et al [33]. These findings highlight the complex and context-specific nature of trust and privacy concerns, suggesting that addressing regional and cultural factors is critical to building user trust in MHAs.

Figures 3 and 4 summarize the relationships between all constructs.

### Quality Appraisal

All included studies were assessed using the relevant JBI critical appraisal tool specific to their methodology, with results indicating overall methodological rigor and low risk of bias [33-46]. Statistical methods were deemed appropriate, although they varied between studies. All cross-sectional studies met key criteria for inclusion, criteria clarity, detailed description of subjects and settings, and valid and reliable outcome measurement [33,35-40,45,46]. Many studies used self-reported AU rather than app usage logs or objective measures [35-37,39,40,45,46], which is suboptimal [10,48] but considered viable given the challenges of collecting AU data in many settings assessed. The quasi-experimental studies typically used a posttest design [34,41-43] and did not include a control group [34,41-44]. However, their methodologies were appropriate given the early-phase and exploratory nature of digital health research. Balki et al [36] conducted a mixed methods study, which was appraised using the JBI checklist for analytical cross-sectional studies for its quantitative component, and the JBI checklist for qualitative studies for its qualitative component. Both components demonstrated methodological rigor and met appraisal criteria.

A full table of quality appraisal findings is presented in Multimedia Appendix 3, detailing results by study and checklist item.

## Discussion

### Principal Findings

In this study, we reviewed 14 publications to identify if TAM and its extensions are able to predict MHA acceptance, highlighting the strongest constructs and extensions. This is the first comprehensive systematic review exclusively assessing the application of TAM for MHAs. We find that TAM and its extensions are effective tools for the assessment of MHA acceptance, able to positively predict the AU of MHAs.

Most studies support the predictive capabilities of TAM [33-40,43,46], and of studies assessing the relationship between BI and AU, most find that BI strongly predicts AU [33-36,38,40]. These findings are concordant with previous literature assessing the validity of TAM in broader contexts, such as health care technology [23-25,49,50]. These results vindicate TAM’s position as the most widely used method of health care technology acceptance evaluation [12,13].

Relationships between core TAM constructs (PU, PEOU, and BI) supported those proposed in the original TAM model [11]. It is widely considered that BI is an excellent predictor of AU; as such, many studies evaluating TAMs stop short of collecting AU data, which can be cumbersome [26,48]. Contrasting some literature, we find that, while BI is a good predictor of AU, the quality of prediction varies [26,41,44,45,48]. Several studies included in this review found that their model inconsistently translated BI to AU [41,44,45], with various factors, including the model itself, affecting this relationship. Explanations for the discrepancy between BI and AU range from framework limitations to the Hawthorne effect and the intention-behavior gap [51,52]. The Hawthorne effect suggests that patients may modify behavior once aware they are being studied [51], hence altering AU of apps and TAM survey responses. The intention-behavior gap proposes three domains explaining the failure to translate BI into AU: (1) failure to initiate, (2) failure to progress toward a goal, and (3) failure to meet a goal [52].

Our findings demonstrate that PEOU and PU remain vital to TAM [33-46] (Figure 2-4). Digital literacy and perception constructs had significant impacts on PEOU [38,41,43,44] (Figures 3 and 4, Table 2). In the context of MHAs, providing users with MHA use training and devices with preloaded MHAs can significantly reduce computer anxiety, while improving self-efficacy and PEOU, helping increase acceptance and AU of technology [38,43-45]. In the consumer technology industry, this approach has already been implemented by industry leaders, acting as both a marketing and support effort [53].

Findings from this review reveal that, while TAM is a good predictor of technology acceptance, broader determinants, beyond PU, PEOU, and BI, must be considered to enhance predictive capabilities [23-25]. Dynamic extended TAM frameworks that adapt to situational and social contexts may further enhance TAM predictive capabilities for MHAs.

Previous literature has proposed a “technology acceptance lifecycle” to assess acceptance and predict BI to continued use of a technology [49]. In this lifecycle approach, technology is deployed; then AU data, alongside TAM constructs, are used to predict BI to continued use [49]. This proposition was refuted by 2 studies in this systematic review that structured their model similarly to this approach but failed to find strong evidence in support [39,42]. While support for the technology acceptance lifecycle [49] was limited, it has introduced a compelling argument for dynamic TAMs, which continually assess the acceptance of MHAs coupled with AU, further enhancing the predictive capabilities.

### Implications of Extended TAM Constructs

We find that the strong predictive capabilities of TAM can be further enhanced by the addition of extended constructs. Our review found that health risk–related constructs conferred substantial improvements to extended TAM models; however, many studies evaluating these constructs were conducted during the COVID-19 pandemic, which may introduce confounding [35-37]. The predictive strengths of social constructs were more heterogeneous (Table 2); hence, the addition of these factors may be more context specific. Digital literacy, trust, and application factor constructs had significant positive effects on extended TAMs. Of note, extensions of TAM hindered the capabilities of some models [41,42,44,45], especially when model relationships are mapped unconventionally or the construct definition or implementation is suboptimal.

The doctor-patient relationship is a critical mediator of application choice and use [41]. Stronger relationships with one’s physicians are associated with increased adherence to medical advice and better general health outcomes [54-56]. Hence, “relationship with doctor” may be a valuable predictive factor for TAM extensions assessing MHAs [41], particularly for apps with high referral rates from HCPs and health authorities, such as MyFitnessPal in the United Kingdom [57-59].

Studies suggested that trust in the app developers is also crucial to acceptance of MHAs [33,35,39]. This is likely due to the sensitive nature of the data collected and processed by MHAs. With the rapidly multiplying number of MHAs available [5,60], trust will likely become an increasingly substantial consideration made by consumers when selecting MHAs. The introduction of MHA accreditation may help to build consumer trust in the MHA market, which is relatively underregulated [5,60].

While regulation is essential to ensure safe and secure MHAs and helps build consumer trust [5,60,61], it is important to avoid a shift toward overregulation [45,61]. McKee et al [45] found that leniency afforded in data protection regulations and compensation structures during the COVID-19 pandemic led to increased use and acceptance of health care–related MHAs to conduct routine psychiatric assessments. While these findings must be considered in the pandemic context, both the PEOU and PU were likely improved, with doctors able to provide care more easily due to the extended scope of regulations, thereby improving accessibility. Previous literature found a similar relationship with stringent data protection regulations hindering digital health uptake more broadly [62-64]. However, this must be balanced with data security and privacy, with other studies suggesting that strong data privacy and security laws were intrinsically linked to consumer trust and MHA use [33,35,37,39]. Ensuring the safety and security of MHAs, enhancing consumer trust and MHA efficacy, while allowing enough leniency to maximize utility, is essential.

The impacts of COVID-19 extended beyond regulation and policy, driving rapid adaptation and uptake of technology, even among the most techno-averse groups [36,61,63,65-67]. These shifts are likely to have long-term effects, driving technology acceptance and reducing technology anxiety, while improving consumers’ PEOU [36,61,65-67]. Moreover, 2 studies found that TAM remained valid throughout the COVID-19 period [35,36]. The pandemic also led to significant increases in perceived health threat and general health risk concerns, which is likely to have driven consumers toward MHAs [35,36].

As MHA adoption continues to grow, physician endorsement should be a focus for regulators. To build trust and drive AU, formal referral and accreditation schemes could be introduced. For example, national bodies might approve MHAs and recommend them for use, allowing physicians to “prescribe” them to certain patients. Such an approach would enhance engagement with high-quality, evidence-based MHAs while leveraging multiple TAM extension domains to maximize MHA acceptance and health outcomes.

The most significant constructs were those assessing the hedonic characteristics of MHAs, with most studies finding them a strong component of their model [36,37,41,42]. Associated with hedonic constructs, app personalization is becoming increasingly essential to consumer engagement and is demanded by the market [68-72]. Personalization may increase enjoyment, PU, and PEOU by tailoring MHAs to the user and their individualized needs. However, in a world of extremely personalized content continuously delivered to consumers, it may act as a deterrent to some users seeking general advice and information, as well as carrying additional risks [39,68,69,73]. This can be addressed by allowing the user to select their desired level of personalization. Features that enhance enjoyment and aesthetic appeal can further improve user engagement, with gamification playing an important role in driving acceptability [42,71].

### Impacts of the Digital Divide

The importance of social inequalities should not be underestimated. Consumers from deprived backgrounds are likely to be less trusting of institutions and app developers, have poorer literacy (both digital and general), and ultimately be most affected by the digital divide [5,37,66,74-78]. These same communities have the greatest burden of disease, more complex care needs, and have risk factors which MHAs are specifically designed to address [5,74,78-80]. This is reminiscent of the inverse care law, which stipulates that the availability of good medical care tends to vary inversely to the demands of the population—being lowest where demand is highest (among deprived populations) [78,80-83]. From a service delivery perspective, due to the higher burden of disease and the substantial care needs in these groups, MHAs offer a unique, cost-effective, and preventative opportunity, ultimately reducing long-term health care expenditure and system burden [3,4,80,84,85].

Our findings suggest that social factors, PU, design aesthetics, and personalization strongly predict the acceptance of MHA, particularly in users from more deprived socioeconomic backgrounds [37]. MHA usefulness, especially, may be higher in deprived communities due to limited access to alternative health management resources relative to those from more secure SES backgrounds [86,87]. The increased predictive capacity of these factors may, in part, be due to greater reliance on community opinions or peer behaviors in decision-making. This suggests a need for designing MHAs that leverage social networks to enhance engagement among these populations. These findings have implications both in terms of their inclusion in extended TAM frameworks and for inclusive app development, promoting user engagement.

To foster consumer trust and engagement, developers and governments could leverage training and support seminars, acting as valuable tools for marketing and support, and building trust, thereby enhancing adoption while minimizing digital literacy barriers, and leveraging social networks and engagement. Furthermore, developers should adopt more inclusive app development processes, guided by the principles established in this review. This should include the integration of enhanced personalization and gamification, which is likely to drive enjoyment, user engagement, and acceptance for all groups. In addition, to generate further enhancements in MHA acceptance, social inequalities in general must be addressed.

On a global scale, this review identified a significant gap in research, with only 1 study conducted in an LMIC [34], and none in LICs. This disparity may be attributed to research barriers in these regions, as well as limited access to technology [88-90]. More studies should be conducted in these regions due to substantial differences in society and culture, technology availability, and the health care challenges faced by their communities. Technology acceptance in these regions likely has different drivers. The potential of MHAs is perhaps even more significant for these regions, where health care access and infrastructure are sparse, due to their cost-effective and efficacious nature [3,4].

Furthermore, future studies should aim to evaluate acceptance during the initial piloting phase, as well as the postadoption phase of MHA implementation, as these stages may reveal distinct drivers of acceptance. In addition, research examining MHA usage among patients with varying health statuses could provide valuable insights into how health conditions impact technology acceptance and inform the development of more inclusive and effective MHAs.

### Limitations

Certain limitations of this systematic review should be acknowledged. First, the included studies were heterogeneous in models, methods, statistical analyses, and outcomes. Several studies relied on self-reported AU data [35-37,39,40,45,46], which, while commonly used in technology acceptance research due to challenges in data collection, may introduce recall bias and reporting inconsistencies [10,48]. Furthermore, many studies recruited predominantly student populations [34,35,39,43,46], and only 1 study was conducted in an LMIC [34], with none in LICs. These factors reflect the current state of research in this field and underscore the need for greater geographic and demographic diversity in future studies to enhance generalizability. Furthermore, English-language restriction potentially excluded valuable insights from non–English-speaking regions where MHAs have gained significant traction, limiting the cultural and geographical scope of our conclusions.

Despite these methodological constraints, our review provides a robust foundation for understanding MHA acceptance through the TAM framework. Our rigorous review process ensures the reliability and validity of our findings, while acknowledging that a larger evidence base would further strengthen the generalizability of conclusions across diverse health care settings and user populations.

Finally, publication bias may have skewed our findings toward positive relationships between TAM variables and mHealth acceptance, as studies reporting significant results are more likely to be published.

### Conclusion

TAM is a robust framework for assessing the acceptance of MHAs. Incorporating social and clinical context-specific extended constructs can significantly enhance TAM’s predictive capabilities. Understanding the interactions between constructs is pivotal for successful integration into TAM extensions. Development of a standardized TAM specification framework and a comprehensive TAM evaluation model, supplemented by an extensive, validated TAM questionnaire bank, will aid in standardization, improving regular assessment, revisions, and refinements to TAM extensions for both MHAs and broader health care technologies. Through continual development and the integration of well-constructed, contextually relevant constructs, alongside informed app development, we can optimize the use of MHAs, ultimately providing a cost-effective and highly efficacious method of health management.

## Appendix

Multimedia Appendix 1 PRISMA Checklist.

Multimedia Appendix 2 Search strategy.

Multimedia Appendix 3 Data extraction and quality appraisal.

## Abbreviations

AU: actual use
BI: behavioral intention
HCP: health care professional
JBI: Joanna Briggs Institute
LIC: low-income country
LMIC: low-middle income country
MHA: mobile health app
mHealth: mobile health
PEOU: perceived ease of use
PRISMA: Preferred Reporting Items for Systematic Reviews and Meta-Analyses
PU: perceived usefulness
SES: socioeconomic status
TAM: technology acceptance model
