# Supplementary material for: Improving Acceptability of mHealth Apps—The Use of the Technology Acceptance Model to Assess the Acceptability of mHealth Apps: Systematic Review
Source: J Med Internet Res. 2025 May 7;27:e66432. doi: 10.2196/66432 (PMC12096023; doi:10.2196/66432)

# **Multimedia Appendix 1: Search Strategy**


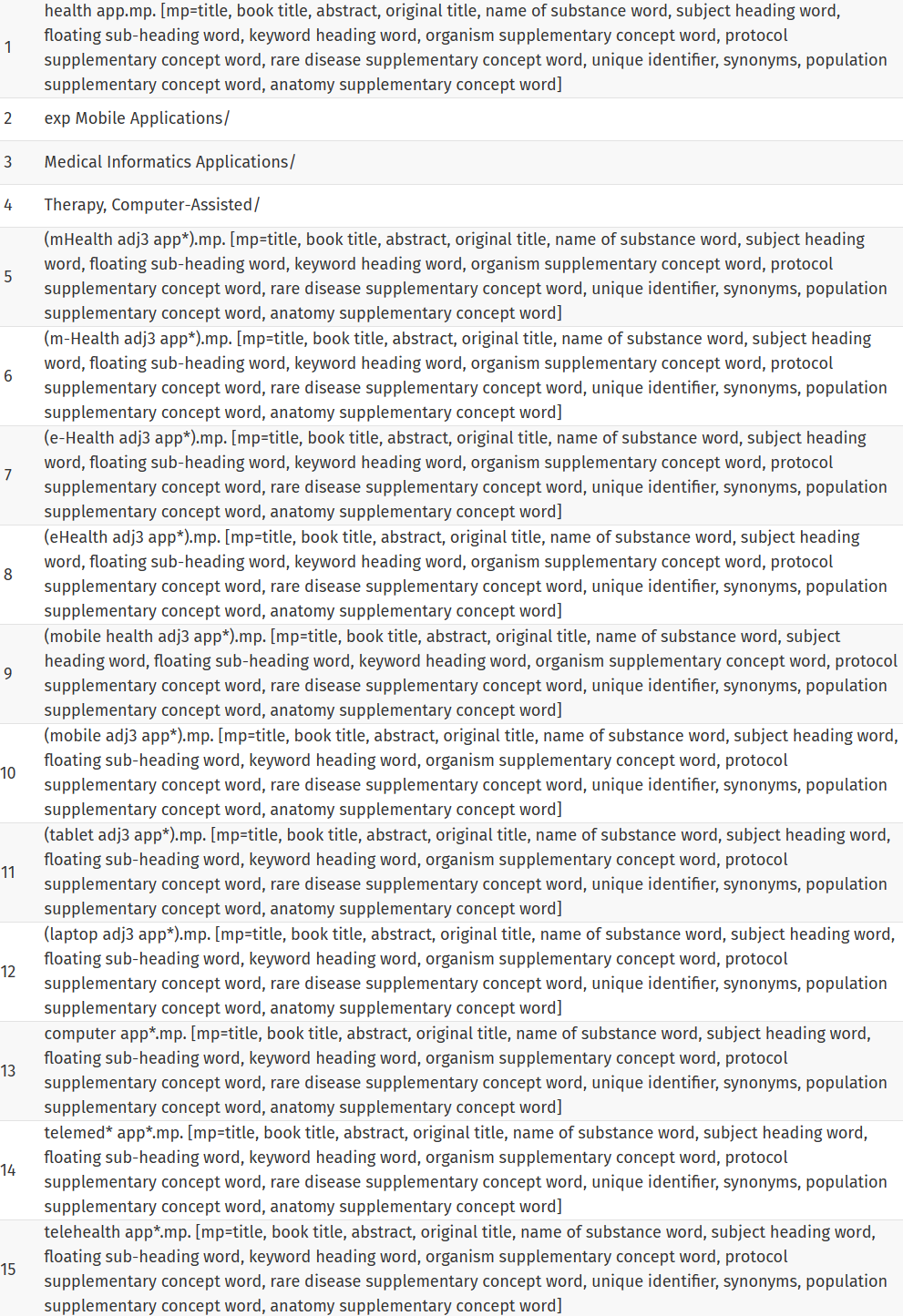

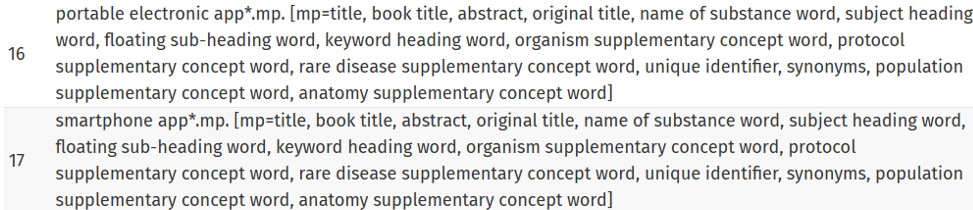


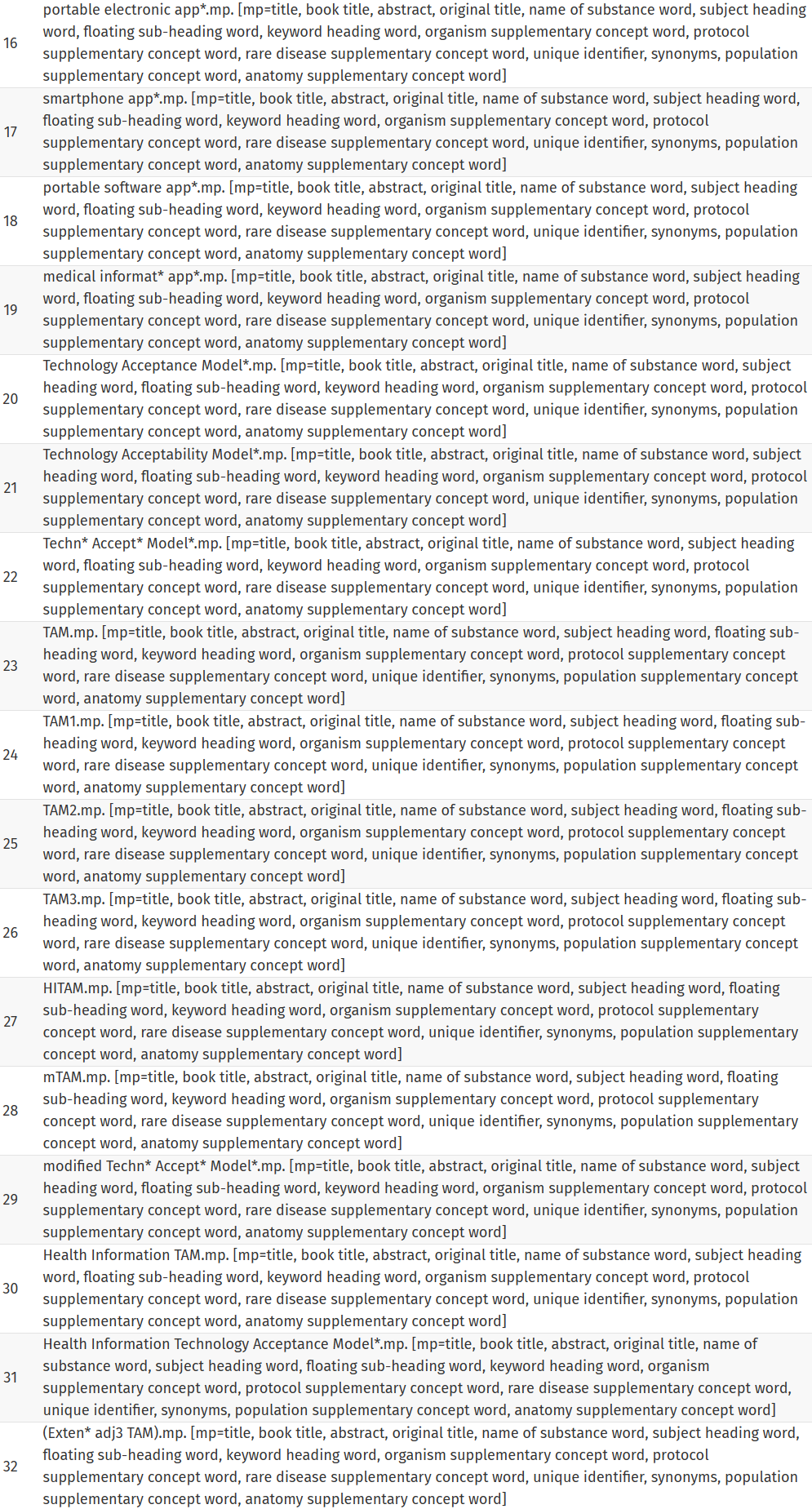


**Figure S1.** Ovid MEDLINE search strategy which was then adapted and applied to all other databases to conduct search.


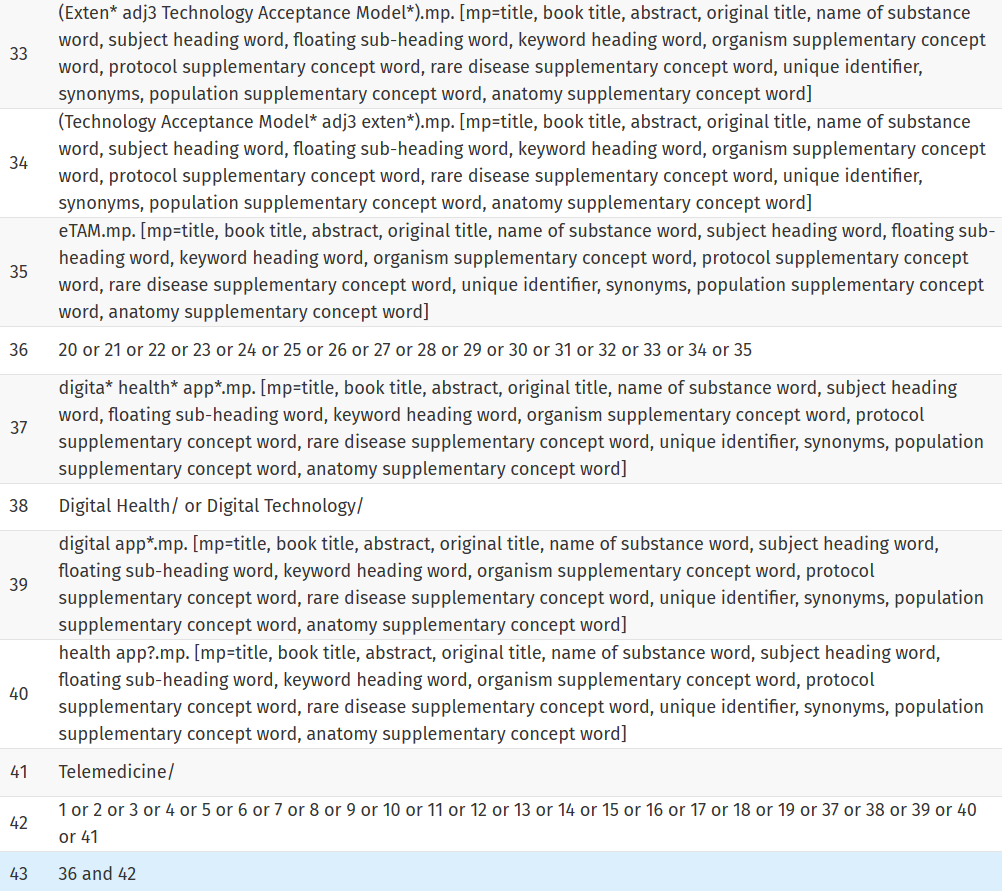

Supplement: Multimedia Appendix 2 [file jmir_v27i1e66432_app2.docx]
