# Supplementary material for: Improving Acceptability of mHealth Apps—The Use of the Technology Acceptance Model to Assess the Acceptability of mHealth Apps: Systematic Review
Source: J Med Internet Res. 2025 May 7;27:e66432. doi: 10.2196/66432 (PMC12096023; doi:10.2196/66432)
Supplement: Multimedia Appendix 3 [file jmir_v27i1e66432_app3.docx]

# **Multimedia Appendix 2: Data Extraction and Quality Appraisal**

| Data Category | Parameter Assessed |
| --- | --- |
| Meta Data | Title |
|  | Authors |
|  | Journal |
|  | Country |
|  | Date of publication |
| Methodology | Study design |
|  | Inclusion criteria |
|  | Exclusion criteria |
|  | Statistical approach |
|  | Sample demographics |
| TAM | MHA used |
|  | MHA clinical domain / use |
|  | TAM model used |
|  | Extensions used |
|  | Justification for extensions |
| Findings | Relationships between model constructs |
|  | AU of MHA |
|  | Strongest construct relationships |

**Table S1**: Data extraction sheet template

| Study ID | Model: Constructs | TAM Assessment Survey and Scales Used | | Overall Evaluation of Technology Acceptance Model Focusing on TAM Constructs (PU, PEOU, BI) | |
| --- | --- | --- | --- | --- | --- |
| Akdur et al. 2020 ^33^ | **TAM**: PU, PEOU, BI,  **Extension**: Price-value, perceived risk, trust | Questionnaire adapted from literature. 5-point Likert scale. | | TAM was effective in predicting user acceptance and predicted actual use moderately well (BI to AU R^2^ = 0.322). PEOU influenced both PU and BI. PU influenced BI. BI was able to predict AU. Perceived risk had no statistically significant relationships. Trust influenced BI. Price-value influenced both PU and BI. | |
| Ali et al. 2021 ^34^ | **TAM**: PU, PEOU, BI | A self-designed questionnaire with a 5-point Likert scale. | | TAM was found to have a relatively strong association between BI → AU → smoking cessation. PEOU was found to influence PU, BI, and AU. PU was related to BI and AU. BI had an impact on AU, which then influenced smoking cessation. The strongest relationships were found between PU and AU in China, and between BI and AU in Pakistan. PEOU was found to influence the most constructs. (PU regression estimate of 0.510 in China and 0.401 in Pakistan.) | |
| Alsyouf et al. 2022 ^35^ | **TAM**: PU, PEOU, BI  **Extension**: Event-related fear, COVID-19 anxiety, trust in government, perceived privacy, social media awareness (SMA), social influence | Survey adapted from literature on a 5-point Likert scale. | | Overall, statistical analysis supported model validity. PEOU was found to influence PU and BI. PU impacted BI. BI impacted AU. Perceived privacy influenced BI. SMA had no statistically significant pathways. Trust in government acted via social influence moderating AU. Event-related fear acted on AU; this relationship was moderated by COVID-19 anxiety. The strongest predictive factor was PEOU. | |
| Balki et al. 2023 ^36^ | **TAM**: PU, PEOU, BI  **Modified TAM**: Perceived Enjoyment (PE)  **Extension**: COVID-19 Anxiety Level, | Survey adapted from literature on a 7-point Likert scale. | | Overall, this study found that TAM remained a valid acceptance predictor, with significant construct relationships even during the pandemic. PU, PEOU, and PE contribute to BI and AU. COVID-19 anxiety had negative relationships with PU, PEOU, PE, BI, and AU. Slight discrepancy between BI and AU (44% had positive BI, but 56% had positive AU). Themes related to acceptance not included in the model but highlighted during qualitative exploration: “influence of the pandemic (situational context)”, privacy, and security. | |
| Bao et al. 2023 ^37^ | **TAM**: PU, PEOU, BI  **Communication Inequality**: design aesthetics, health valuation, hedonic motivation, trust, self-efficacy, descriptive norms, injunctive norms | Survey derived from literature. | Partial support for model. Integration of communication inequality aided in the model assessment. Only relationships assessed were from constructs to BI and AU. PU was associated with BI but not AU. PEOU had no significant relationships. Design aesthetic associated with BI but not AU. Health valuation no significant associations. Hedonic motivation associated with BI and AU. Descriptive norms associated with AU. Injunctive norms had no statistically significant relationships. Trust had no significant relationships. Education was weakly associated with AU. PU and design aesthetic were found to narrow the BI gap between education levels. Descriptive and injunctive norms were also found to narrow this gap. PU was the strongest predictor of the TAM constructs, and hedonic motivation’s association with BI and AU was the strongest overall. | |  |

| Byrd IV et al. 2021 ^38^ | **TAM**: PU, PEOU, BI  **Diffusion of Innovation Theory**: Personal Innovativeness in the Domain of Information Technology (PIIT), Perceived Critical Mass (PCM) | Survey items adapted from literature. 5-point Likert scale used. | Model explained substantially more variance in BI and AU than the literature (limited use = 61%, expanded use = 72%, literature = 40-70%). Population stratified into two groups – expanded use (devices were provided), and limited use (no device was provided). PEOU was found to influence PU and BI. The effect of PEOU on BI was greater than its effects on PU. Effects of PEOU on BI were not significant in the expanded use group. PU influenced BI. Large effect of BI on AU. BI explained 20% more AU variance in limited use group. PIIT had no relationship with PU. PIIT had a small positive impact on PEOU and BI in both groups. PCM influenced PEOU, PU, and BI in both groups.  The strongest relationship in both groups was that of PEOU on PU. |
| --- | --- | --- | --- |
| Cho et al. 2015 ^39^ | **TAM**: PU, PEOU, BI  **Displacement Theory** and **Complementarity Theory**: Perceived Credibility of Mass Media (PCMM), Perceived Credibility of Online Media (PCOM) | Survey modified from literature, scaled on a 5-point Likert-type scale. | Overall, the study found significant relationships and supported this TAM. PEOU influenced BI. PU influenced BI. PU mediated the effects of PEOU on BI. PCMM influenced PU and PEOU. PCOM had no significant relationships. |
| Cramer et al. 2022 ^40^ | **TAM**: PU, PEOU, BI | Self-developed questionnaire on a 7-point Likert scale. | Model supported the use of TAM. TAM core construct relationships were confirmed. PEOU impacted PU and BI. PU impacted BI. BI impacted AU. Additional indirect pathways assessed were also statistically significant. Model was more significant among sexual minority groups than the heterosexual group. Of note, the heterosexual group was substantially smaller and may be limited by statistical power. |
| Dou et al. 2017 ^41^ | **TAM**: PU, PEOU, BI  **TAM2**: Social Influence, Usage Experience  **Health-Belief Model**: Perceived Health Threat (PHT), Self-Efficacy  **Dual-Factor Model**: Resistance to Change (RTC)  **Extension**: Relationship with Doctor (RWD), Demographics | Survey items derived from literature. A 5-point Likert scale was used. | Model and BI to had relatively weak predictive capability explaining only 41% of the variance in BI (R^2^ = 0.412), and 11% of the variance in AU (R^2^ = 0.111). PEOU influenced PU, but not BI. PU influenced BI. BI had a statistically significant relationship with AU. RTC had a significant negative relationship with BI.  PHT had significant positive relationships with BI and PU. RWD had significant positive influences on PU and PEOU, with a significant negative impact on RTC. Social influence had no significant relationships. Usage experience and self-efficacy has significant positive influences on PEOU. The model found that BI was influenced by perceived health threat, RTC, and PU. The strongest relationship was that between PU and BI. |
| Hurmuz et al. 2022 ^42^ | **TAM**: PU, PEOU, BICU  **Extension**: Enjoyment, aesthetics, and control | Survey based on past literature on a 7-point Likert scale. | Model was a poor predictor. Hypothesised pathways were unconventional. Many pathways validated by previous studies were found to be redundant. Only statistically significant pathways were: PU → BICU, PEOU → use duration and frequency, Enjoyment → PU, Aesthetics → PEOU. The largest effect size was between PU → BICU (1.498) |

| Jeon et al. 2015 ^43^ | **TAM(2)**: PU, PEOU, BI  **Innovation Diffusion Theory**: Compatibility,  **Extension**: Self-Efficacy, Technical Support and Training | Literature derived survey, on a 5-point Likert scale. | No definitive conclusion on the model, but model fit was good, and relationships were statistically significant. PEOU elicited positive impacts on PU and BI. PU had a significant relationship with BI. Technical support and training positively affected PEOU. Compatibility positively influenced PU, PEOU, and BI. Self-efficacy did not have any significant relationships, but was affected by compatibility and technical, support and training. PU was the most significant variable in this model. |
| --- | --- | --- | --- |
| Louissaint et al. 2020 ^44^ | **TAM**: PU, PEOU, BI  **Extension**: Computer Anxiety  *Additional analysis includes: presence of a care giver, presence of an ascites medication.* | Survey was self-designed survey and adapted from literature, responses graded on a 7-point Likert scale. | Good BI predictive capabilities with 64% of variance in BI explained. The model could not predict AU. PU had a relationship with BI. PEOU was related to BI. Computer anxiety was related with PEOU. Presence of a care giver was associated with improved PEOU, PU, and reduced computer anxiety. Increasing age was associated with increasing computer anxiety. MHA download was associated with PU, PEOU, computer anxiety, and BI. “Computer anxiety was the only variable significantly associated with willingness to download”. No associations of any construct or demographic factor with AU.  Patients completed questionnaire prior to app use hence authors suggest that findings of this study are applicable to all application designed for cirrhosis and many for liver disease |
| McKee et al. 2021 ^45^ | **TAM**: PU, PEOU, BI  **TRA**: Attitude, Subjective Norms | Literature derived validated questionnaire with 7-point Likert scale. | Combined TRA and TAM model explained 14.5% of AU variance. TAM explained 33% AU variance. TRA 34.1% AU variance. **TRA model**: subjective norms had effects on BI and AU. attitude towards telepsychology was associated with BI. BI was associated with AU. In the **TAM model**: PU had significant relationships with attitude and BI, PEOU had effects on attitudes towards telepsychology and BI, attitude had relationships with BI, BI was significantly associated with AU, indirect effects of constructs were also found PEOU → attitude → BI → AU, PEOU → BI → AU, PU → attitude → BI → AU. **Combined** TAM and TRA model (BI was removed): Model fit indices were positive overall but not unanimously, with RMSEA and TLI indicating poor fit, subjective norms had relationships with PU and PEOU, PU had direct effects on AU, PEOU had direct effects on AU. In TRA attitudes was the strongest predictor of BI. In TAM, PEOU was the strongest predictor of BI. In the combined TAM and TRA model, subjective norms was the strongest predictor of PU. |
| Shemesh et al. 2020 ^46^ | **TAM**: PU, PEOU, BI  **Extension**: Attitudes | Self-developed questionnaire with 5-point Likert scale. | Strong for TAM. Model explained 51% of variance in BI. Among the complete study population, validate all TAM pathways and attitudes to have effects on BI. PEOU and attitudes varied significantly between MHA users and non-users. MHA users: PU, PEOU, and attitudes had significant relationships with BI. MHA non-users: the only statistically significant construct was attitudes. Attitudes was strongest predictor of BI explaining 30% of variance alone. |

**Table S2**. Technology acceptance model evaluation table highlighting the key constructs evaluated by the study and the strengths of relevant construct relationships.


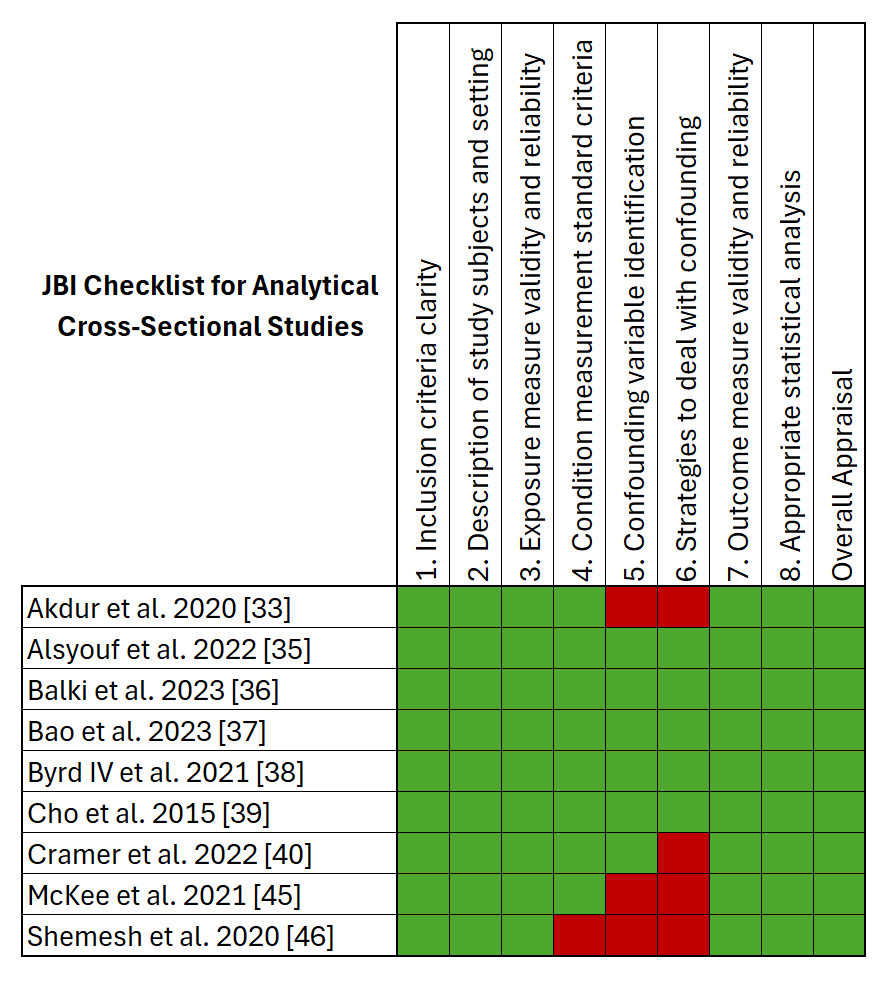

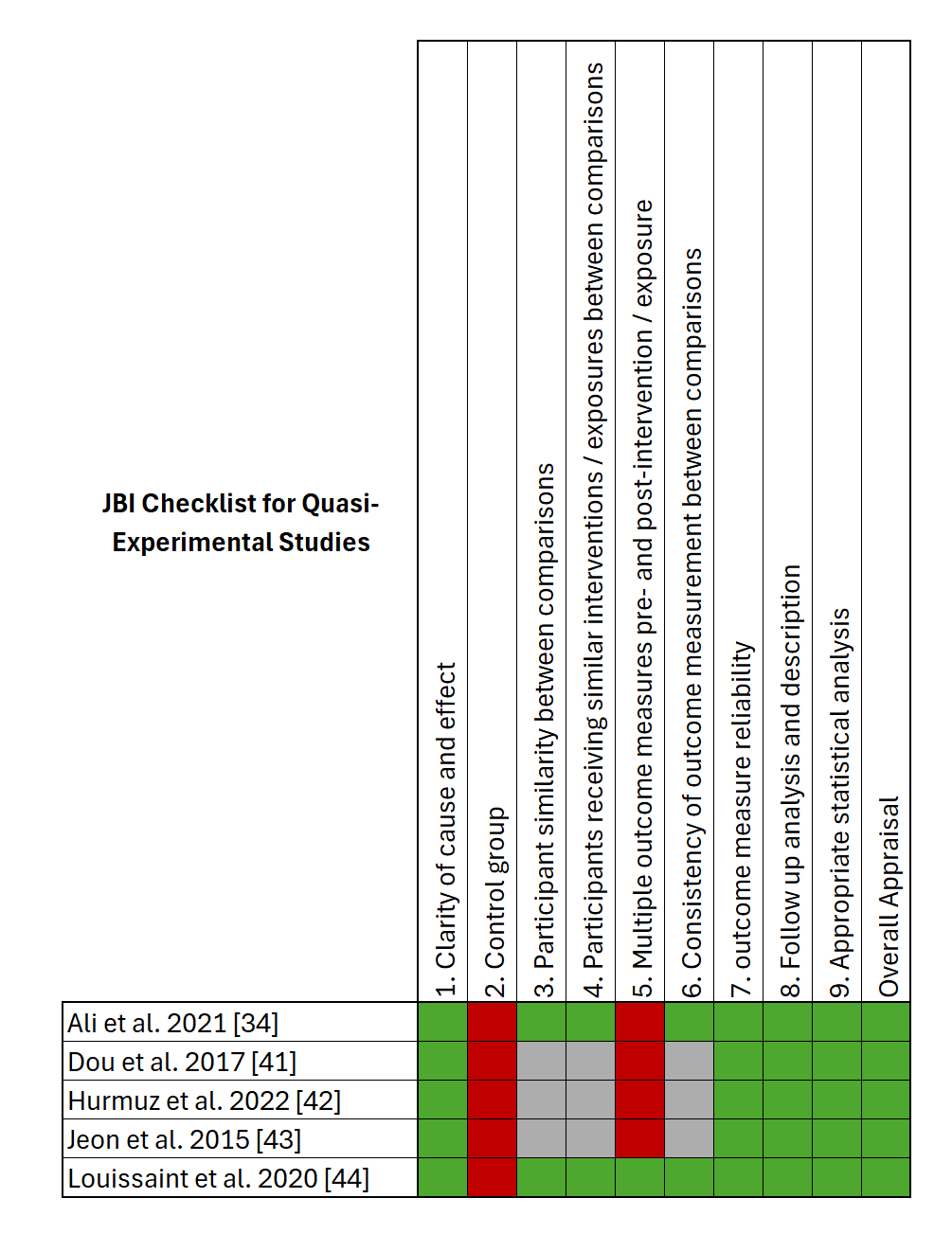

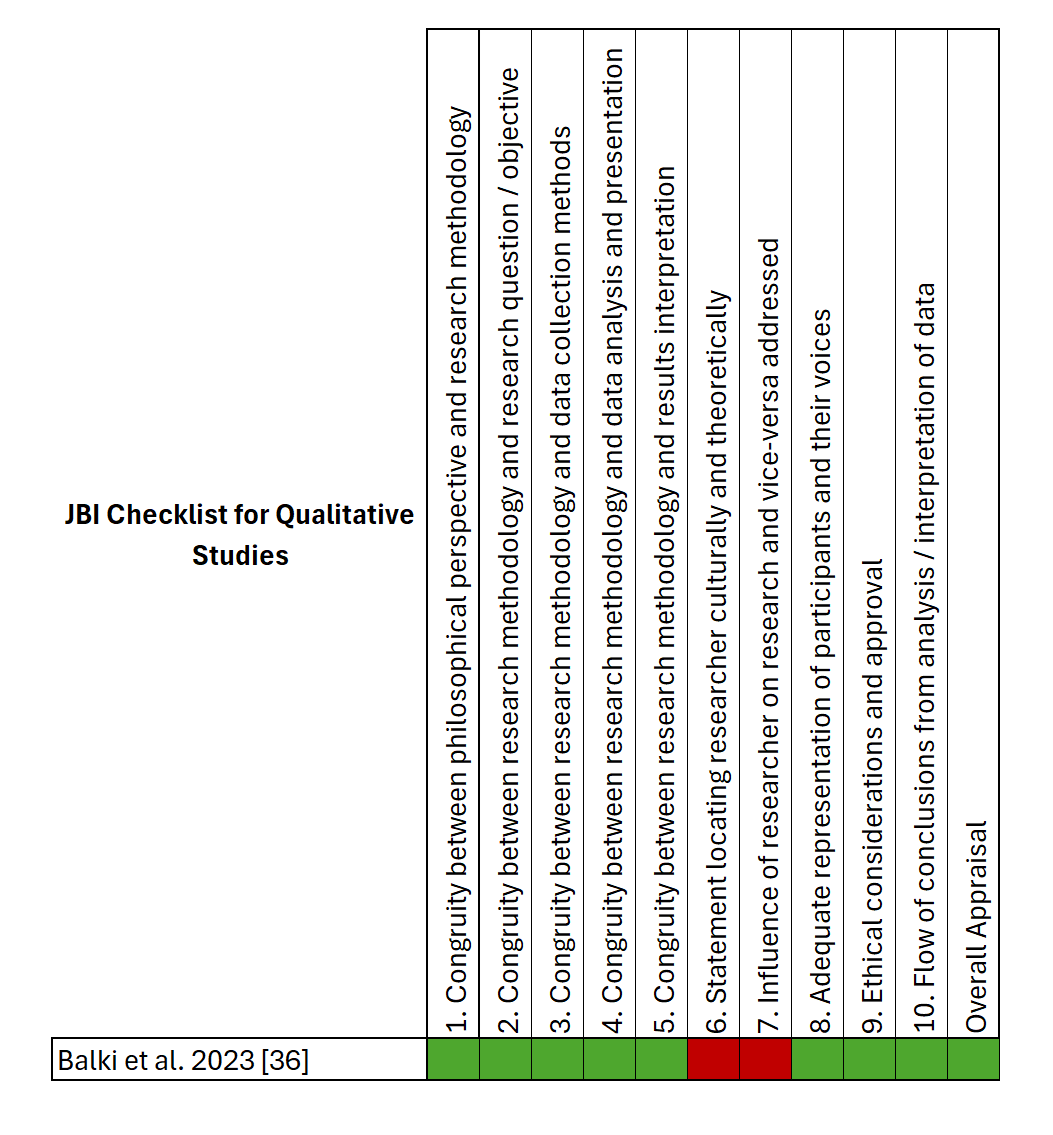


**Figure S1.** Quality assessment tables of all papers included in this review. Green = Yes, Red = No, Grey = N/A

| Abbreviation | Full Form |
| --- | --- |
| AI | Artificial Intelligence |
| AU | Actual Use |
| BI | Behavioural Intention (to use application) |
| EHRs | Electronic Health Records |
| genAI | Generative Artificial Intelligence |
| HBM | Health Belief Model |
| HCP | Healthcare Professional |
| HITAM | Health Information Technology Acceptance Model |
| JBI | Joanna Briggs Institute |
| MHA | Mobile Health Applications |
| mHealth | Mobile Health |
| PCM | Perceived Critical Mass |
| PCM | Perceived Critical Mass |
| PCMM | Perceived Credibility of Mass Media |
| PCOM | Perceived Credibility of Online Media |
| PE | Perceived Enjoyment |
| PEOU | Perceived Ease of Use |
| PHT | Perceived Health Threat |
| PIIT | Personal Innovativeness in the Domain of Information Technology |
| PU | Perceived Usefulness |
| RTC | Resistance To Change |
| RWD | Relationship With Doctor |
| SMA | Social Media Awareness |
| TAM | Technology Acceptance Model |
| TPB | Theory of Planned Behaviour |
| TRA | Theory of Reasonable Action |

**Table S3.** Abbreviations
